# A Machine Learning Approach to Predict Remission in Patients With Psoriatic Arthritis on Treatment With Secukinumab

**DOI:** 10.3389/fimmu.2022.917939

**Published:** 2022-06-27

**Authors:** Vincenzo Venerito, Giuseppe Lopalco, Anna Abbruzzese, Sergio Colella, Maria Morrone, Sabina Tangaro, Florenzo Iannone

**Affiliations:** ^1^Rheumatology Unit, Department of Emergency and Organ Transplantations, University of Bari “Aldo Moro”, Bari, Italy; ^2^Dipartimento di Scienze del Suolo, della Pianta e degli Alimenti, University of Bari “Aldo Moro”, Bari, Italy; ^3^Istituto Nazionale di Fisica Nucleare - Sezione di Bari, Bari, Italy

**Keywords:** psoriatic arthritis (PsA), machine learning, secukinumab, fibromyalgia (FMS), axial

## Abstract

**Background:**

Psoriatic Arthritis (PsA) is a multifactorial disease, and predicting remission is challenging. Machine learning (ML) is a promising tool for building multi-parametric models to predict clinical outcomes. We aimed at developing a ML algorithm to predict the probability of remission in PsA patients on treatment with Secukinumab (SEC).

**Methods:**

PsA patients undergoing SEC treatment between September 2017 and September 2020 were retrospectively analyzed. At baseline and 12-month follow-up, we retrieved demographic and clinical characteristics, including Body Mass Index (BMI), disease phenotypes, Disease Activity in PsA (DAPSA), Leeds Enthesitis Index (LEI) and presence/absence of comorbidities, including fibromyalgia and metabolic syndrome. Two random feature elimination wrappers, based on an eXtreme Gradient Boosting (XGBoost) and Logistic Regression (LR), were trained and validated with 10-fold cross-validation for predicting 12-month DAPSA remission with an attribute core set with the least number of predictors. The performance of each algorithm was assessed in terms of accuracy, precision, recall and area under receiver operating characteristic curve (AUROC).

**Results:**

One-hundred-nineteen patients were selected. At 12 months, 20 out of 119 patients (25.21%) achieved DAPSA remission. Accuracy and AUROC of XGBoost was of 0.97 ± 0.06 and 0.97 ± 0.07, overtaking LR (accuracy 0.73 ± 0.09, AUROC 0.78 ± 0.14). Baseline DAPSA, fibromyalgia and axial disease were the most important attributes for the algorithm and were negatively associated with 12-month DAPSA remission.

**Conclusions:**

A ML approach may identify SEC good responders. Patients with a high disease burden and axial disease with comorbid fibromyalgia seem challenging to treat.

## Introduction

Psoriatic Arthritis (PsA) is a heterogeneous condition in its clinical presentation and disease course. Immune dysregulation, with altered cytokines expression and cellular phenotypes, is responsible for the typical clinical features of PsA, which involve both peripheral joints and the axial skeleton (1, 2). In addition to such manifestations, patients with PsA can often suffer from extra-articular manifestations, which are genetically and immunologically correlated to these features and include psoriasis, inflammatory bowel diseases, and uveitis (3, 4). PsA pathogenesis is complex, with nonlinear interaction between genetic and environmental factors, such as obesity and trauma, triggering the preclinical activation of the immune system in patients with psoriasis, mainly involving the activation of the interleukin (IL-)23–IL-17 axis, (2). By interfering with such a pathway, novel therapies with biotechnological agents enable the complete clearing of psoriasis in most patients but a PsA good control in only half the patients (2). Hence, profiling PsA patients more likely to benefit from IL-17 blockade is an unmet clinical need.

Secukinumab (SEC), a human monoclonal antibody that directly inhibits interleukin-17A, has demonstrated efficacy in patients with PsA in phase III FUTURE studies (5), but only sparse data exist about predictors of disease remission in PsA patients in real-life settings (6, 7). While logistic regression (LR) was the algorithm of choice to find independent predictors in multivariable models, it must be noted that in previous real-life studies, the hypotheses were usually based on the unreal assumption that the association between the prognostic factors and PsA remission is direct and isolated (8–10).On the contrary, LR is not suitable for modelling non-independent variables (6), being inadequate to explicitly describe the complex relationship between prognostic factors and remission for complex multifactorial diseases such as PsA. Additionally, previous multivariable models often lacked rigorous internal and external validation (11, 12), leaving internal consistency unchecked and raising doubts on model validity in the general PsA population.

Machine learning (ML) is emerging as a promising tool for implementing complex multi-parametric decision algorithms. Supervised ML algorithms have proven effective in predicting treatment responses and disease progression in patients with rheumatic diseases and can handle complex, non-linear relationships between patient attributes that are difficult to model with traditional statistical methods (13, 14). Therefore, this study aimed to assess whether a ML approach may be useful to identify those patients more likely to achieve disease remission on SEC.

## Materials and Methods

### Data Gathering

Patients with classified PsA according to CASPAR criteria referred to a tertiary centre who underwent Secukinumab therapy from September 2017 to December 2020 with at least 12 months of continuous treatment were included in the analysis. Demographic (age and gender), laboratory (Erythrocyte Sedimentation Rate (ESR), C Reactive Protein (CRP) serum level) and clinical characteristics were retrospectively gathered either at baseline, and 12-month follow up. We recorded the presence/absence of comorbidities [fibromyalgia (FMS) and metabolic syndrome (MetSyn)], Body Mass Index (BMI), disease duration, treatment line, disease domain (oligoarthritis VS polyarthritis, axial involvement), Disease Activity Index in Psoriatic Arthritis (DAPSA), Leeds Enthesitis Index (LEI). Bath Ankylosing Spondylitis Activity index (BASDAI), Psoriasis Area Severity Index (PASI), concomitant conventional synthetic anti-rheumatic drug (csDMARDs) and Health Assessment Questionnaire Disability Index (HAQ-DI). Axial involvement was defined as the presence of radiographic sacroiliitis (as for clinician and/or radiologist judgement) and/or MRI inflammatory changes to the spine and/or sacroiliac joints, both in the presence of inflammatory pain. The study was approved and reviewed by the local Ethical Committee (Biopure registry, IRB Approval n.5940, Azienda Ospedaliera Universitaria di Bari). This study followed STARD guidelines and the TRIPOD statement. All patients provided written informed consent.

### Outcome of Interest

The predictive modelling analysis aimed at forecasting the probability of DAPSA remission at 12 months from Secukinumab onset, represented as a Boolean variable.

### Traditional Statistics

Student’s t-test or Mann Whitney U test was used as appropriate to identify differences among continuous variables between groups. χ^2^ test was used to determine the difference among Boolean variables in a contingency table. The significance level at α=0.05 was used.

### Attributes Selection

For classification with small training samples and high dimensionality, feature selection plays an essential role in avoiding overfitting and improving classification performance. One commonly used feature selection method for small samples problems is the wrapper feature selection using the recursive feature elimination (RFE) algorithm (1). RFE needs an algorithm to be embedded. Provided with a model with feature coefficients (e.g. regression) or importance factors (e.g. tree algorithms), RFE starts from all features and gradually eliminates the least important feature. Once all features are removed, the algorithm returns the subset that gives the best performance (*backward selection)*. RFE can generate different subsets of features based on various criteria. The subgroup generated in each step will be used to build a model and train the learning algorithm iteratively.

This is achieved by fitting the given machine learning algorithm used in the RFE core, ranking features by importance, discarding the least important features, and re-fitting the model (**Supplementary Material**). We point out that, by design, the RFE algorithm never relies on validation data to achieve this result. We repeated this process five times using different random seeds to check feature stability for each algorithm. Most often selected attributes were considered part of the final attribute core set (**Supplementary Material**). The analysis was implemented in a Python 3.9 environment using scikit-learn (ver. 0.22.1) and XGBoost (ver. 1.1.0) libraries (12). Two different linear and nonlinear classifiers were used to train and validate the RFE with 10-fold cross-validation for predicting DAPSA remission. For further details regarding cross-validation, see **Supplementary Material**. For linear modelling, an LR was used; for nonlinear modelling, a decision tree-based algorithm, namely *extreme gradient boosting* (XGBoost) (15), was tested. A repeated grid search with cross-validation was used for optimal hyperparameter tuning to maximize the classifiers’ performance (**Supplementary Material**) (16). For each classifier, we plotted ROC curves, and then AUROC was determined. Then, based on the optimal probability cut-off [Youden’s Index (17)], classifiers’ performance was compared with the following metrics:


$$\text{Accuracy}=\frac{truepositives+truenegatives}{truepositives+truenegatives+falsepositives+falsenegatives}$$

$$\text{Recall}\;\;=\frac{truepositives}{truepositives+falsenegatives}$$

$$\text{Precision}=\frac{truepositives}{truepositives+falsepositives}$$

Most ML methods are often referred to as *black boxes* for the complexity of the underlying mechanism. In recent years efforts to improve ML explainability have been made. XGboost methods allow us to obtain the relative importance of tshe prediction of each attribute. For explainability, we plotted the relative importance of each feature, including the training core set. We also determined the odds ratio (OR) for predictors included in the LR model.

### Probability Calibration

A classification model generally forecasts a binary outcome for a given observation and class. In predicting, a model may output the probability of an observation belonging to each possible class (13). This case provides some flexibility in the way predictions are interpreted and presented, allowing the choice of a threshold, as mentioned above, Youden’s index. For a model to be reliable, the estimated class probabilities should reflect the actual underlying probability of the sample. A diagnostic calibration curve for the candidate best classifier was also plotted to check these assumptions, and, consequently, 10-fold isotonic calibration was carried out.

## Results

One-hundred-nineteen patients (female 66/119, 55.46%) with mean age ( ± SD) of 52.74 ± 10.37 and a mean disease duration of 7.41 ± 4.44 years underwent SEC treatment during the observation period. Most of them were a 3^rd^ treatment line or beyond. In 91.60% of them (109/119), SEC was prescribed at 300 mg/4 weeks. Combined therapy with csDMARDs was prescribed in 74 out of 119 patients (62.18%); 54 out of 119 (45.38%) were on concomitant steroid therapy. The most prevalent clinical phenotype was oligoarticular disease (74.79%, 89/119), whereas 41 out of 119 had the concomitant axial disease (34.45%). Skin psoriasis was evident in 107 patients (89.92%). Mean BMI was 27.61 ± 5.31, with MetSyn diagnosed in more than half of the cohort (69/119, 57.98%). At baseline, comorbid FMS was present in 24 out of 119 of them. For complete patient characteristics, see **Table 1**.

**Table 1:** Patient Characteristics.

Patient Characteristics	Secukinumab Baseline			12-month follow-up		
	Av.Obs.			Av.Obs		
Age, mean (SD)	117	52.74	10.37			
Female, n (%)	119	66	55.46			
BMI, years, mean (SD)	119	27.61	5.31			
Disease duration, years, mean (SD)	119	7.41	4.44			
Axial Disease, n (%)	119	41	34.45			
Active Dactylitis, n (%)	119	37	31.09			
Active Psoriasis, n (%)	119	107	89.92			
Polyarticular, n (%)	119	30	25.21			
CRP, mg/L, mean (SD)	113	7.77	15.09	113	5.59	5.52
ESR, mm/h, mean (SD)	115	17.34	15.53	115	16.82	12.57
MetSyn, n (%)	119	69	57.98			
FMS, n (%)	119	24	20.17			
DAPSA, mean (SD)	119	16.80	9.65	119	9.60*	7.80
DAPSA Remission, mean (SD)				119	30	25.21
LEI, median (IQR)	114	0	0-1	112	0**	0-0
HAQ-DI, mean (SD)	117	1	0.07	117	0.98	0.07
HAQ-DI improvement ≥ 0.35, n (%)				117	22	18.49
PASI, mean (SD)	119	2.22	3.11	119	0.84*	1.74
BASDAI, mean (SD)	41	4.60	2.07	41	3.80***	1.97
Steroid, n (%)	119	54	45.38	119	25****	21.01
Combotherapy, n (%)	119	74	62.18			
Methotrexate, n (%)	74	57	77,0			
Sulfasalazine, n (%)	74	13	17.57			
Leflunomide, n (%)	74	4	5.40			
bDMARD treatment 1st line, n (%)	119	27	22.69			
bDMARD treatment 2nd line, n (%)	119	29	24.37			
bDMARD treatment 3rd line and beyond, n (%)	119	63	52.94			
Secukinumab 300mg/4 weeks, n(%)	119	109	91.60			

Av.Obs., Available Observations; BASDAI, Bath Ankylosing Spondylitis Disease Activity Index; bDMARD, biologic disease-modifying anti-rheumatic drugs; BMI, Body Mass Index; CRP, C Reactive Protein; DAPSA, Disease Activity in Psoriatic Arthritis; ESR, Erythrocyte Sedimentation Rate; FMS, Fibromylagia; HAQ-DI, Health Assessment Questionnaire- Disability Index; LEI, Leeds Enthesitis Index; MetSyn, Metabolic Syndrome; PASI, Psoriasis Area Severity Index; SD, Standard Deviation.

*<0.0001.

**0.0008.

***0.0006.

****0.0001.

At SEC baseline, DAPSA was 16.80 ± 9.65, whereas mean PASI was 2.22 ± 3.11; median (IQR) LEI was 0 (0–1), whereas patients with axial involvement scored a mean BASDAI of 4.60 ± 2.07. Regarding patient-reported outcomes (PROs), HAQ-DI was 1 ± 0.07.

At 12-month follow-up, most of the clinimetrics significantly improved, with mean DAPSA decreasing to 9.60 ± 7.80 (p<0.0001), median LEI to 0 (0-0) (p<0.0001), mean PASI to 0.84 ± 1.74 (p<0.0001) and mean BASDAI to 3.80 ± 1.97 (p<0.0006). Of note, SEC had a steroid-sparing effect also, as only 25 out of 119 patients (21.01%, p=0.0001) were still on steroids at 12 months. Twenty out of 119 patients (25.21%) achieved DAPSA remission, whereas 22 patients (18.49%) achieved a HAQ-DI improvement ≥ 0.35. For full clinimetrics at 12-month follow up, see **Table 1**.

In general, SEC was well tolerated, with 4/119 patients (3.36%) complaining of upper respiratory tract infections, not requiring treatment discontinuation and 3/119 patients (2.52%) developed rash and pruritus at the injection site. No patients complained of gastrointestinal symptoms suggestive of active inflammatory bowel disease throughout the observation period.

**Figure 1:**
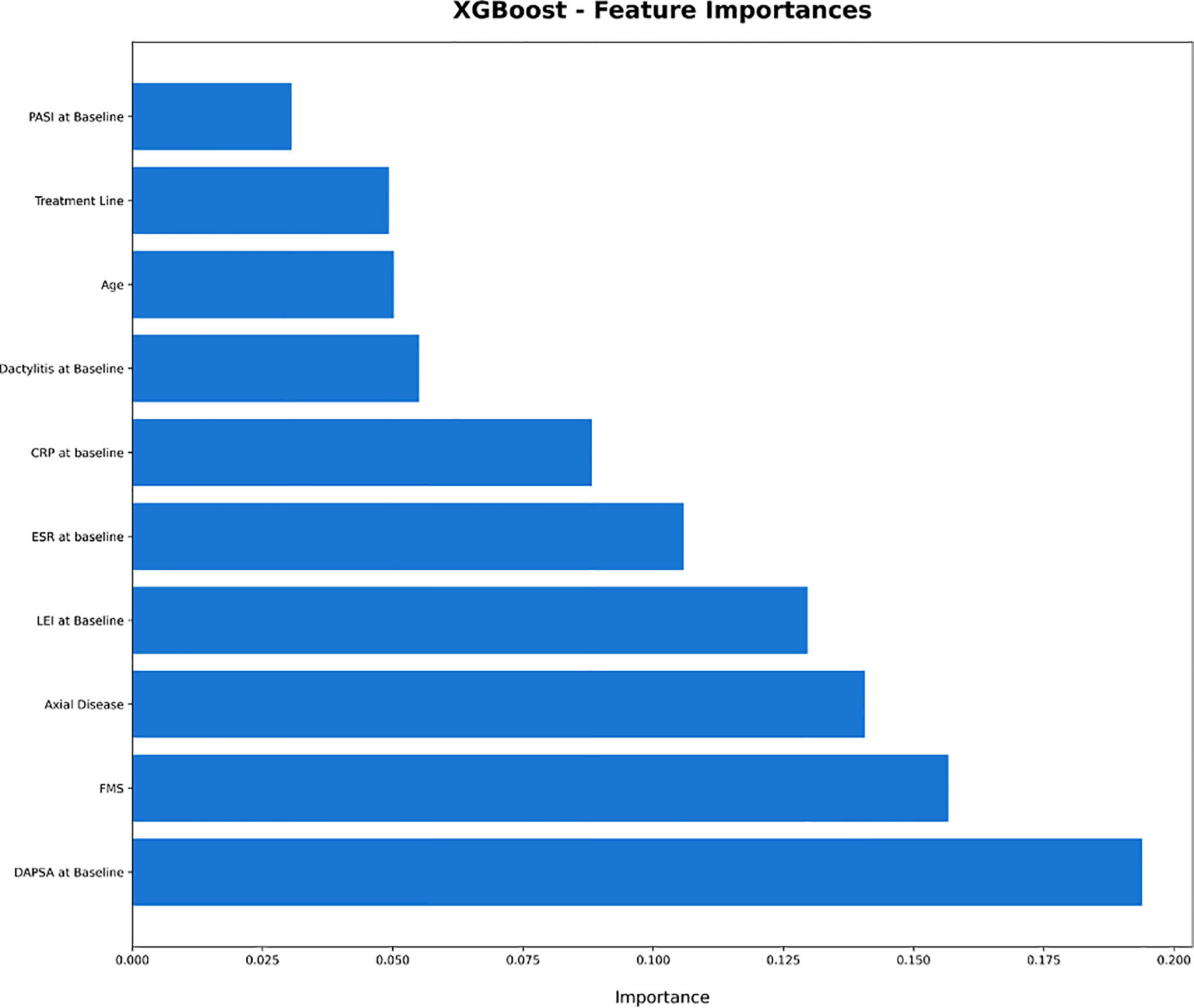
Plot of the feature importance of the attribute core set of eXtreme Gradient Boosting. CRP, C Reactive Protein; DAPSA, Disease Activity in Psoriatic Arthritis; ESR, Erythrocyte Sedimentation Rate; FMS, Fibromyalgia; HAQ-DI, Health Assessment Questionnaire- Disability Index; LEI, Leeds Enthesitis Index; PASI, Psoriasis Area Severity Index; XGBoost: eXtreme Gradient Boosting.

**Figure 2:**
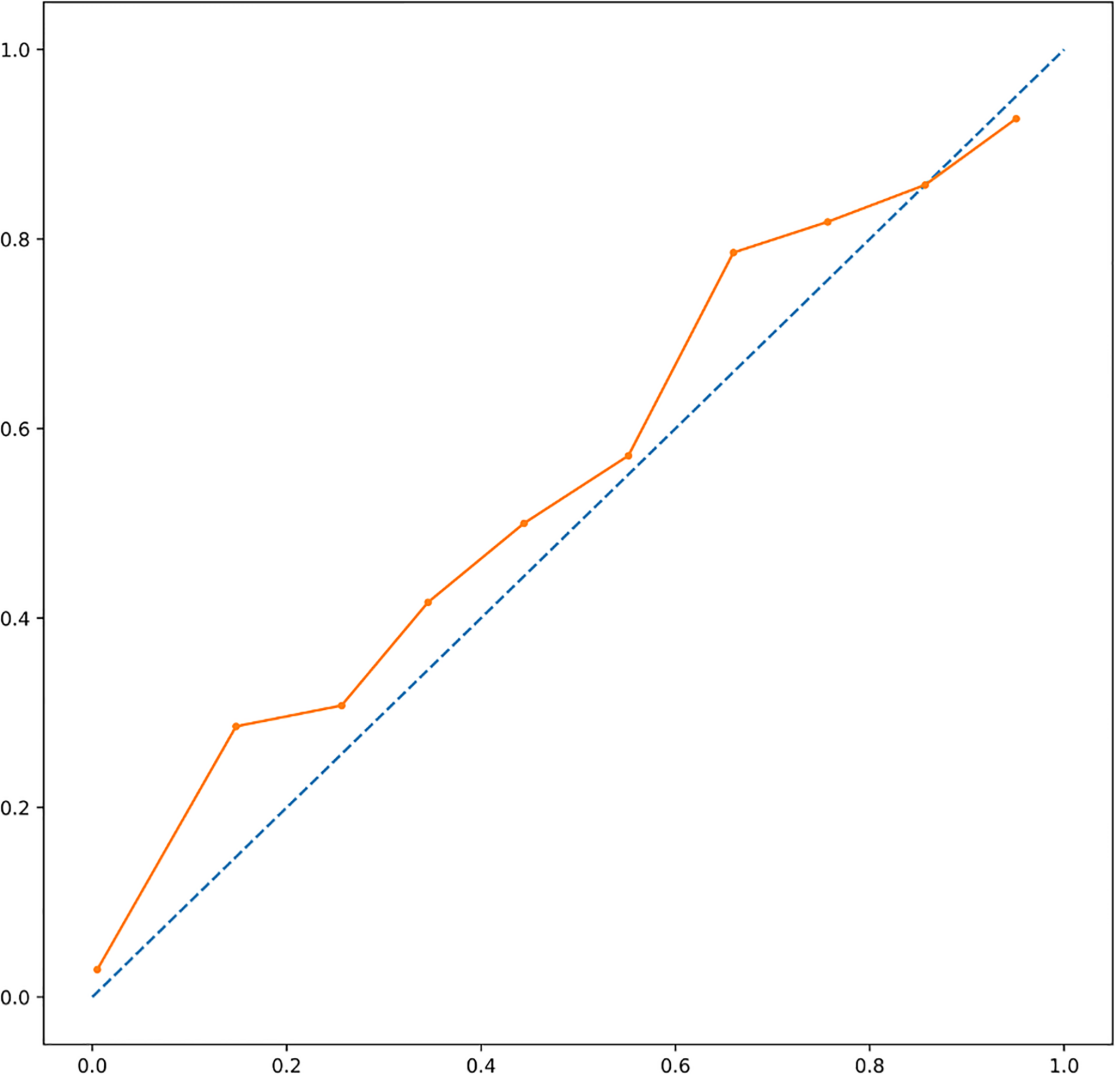
Area under receiver operating characteristic curve of the algorithms. Left panel, eXtreme Gradient Boosting. Right Panel, Logistic Regression.

**Figure 3:**
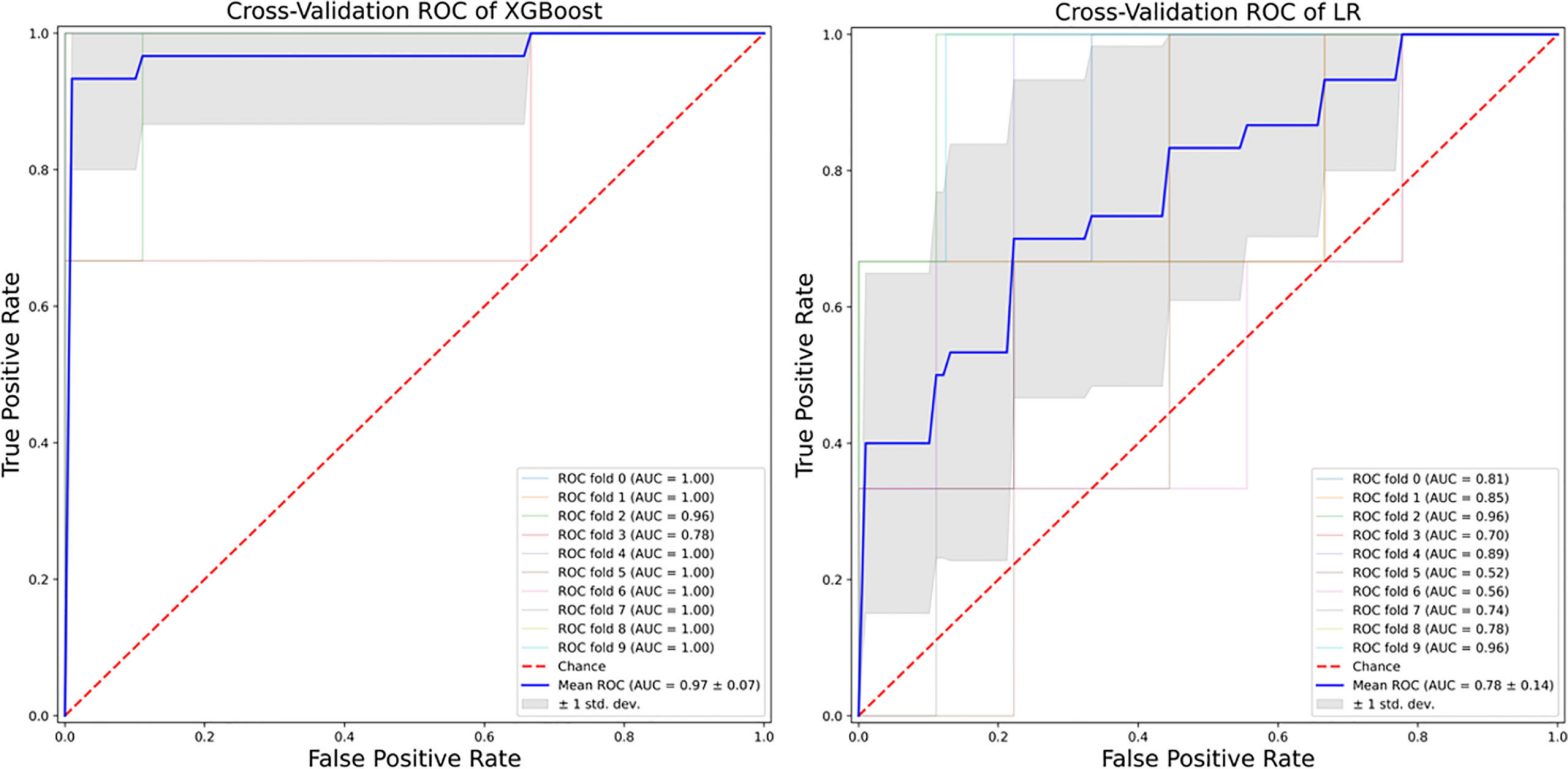
A diagnostic calibration has been plotted for XGBoost after 10-fold isotonic calibration; DAPSA remission roughly happened with an observed relative frequency consistent with the forecast value, showing a suitable calibration curve. DAPSA, Disease Activity in Psoriatic Arthritis; XGBoost, eXtreme Gradient Boosting.

XGBoost-based RFE attribute core set included DAPSA at baseline, presence/absence of FMS, axial Disease, LEI, CRP (mg/l) and ESR (mm/h) at baseline, presence/absence of dactylitis at baseline, age, treatment line and PASI at baseline, listed form the most to the least important. In **Figure 1**, attributes were plotted according to their importance for ML prediction. Conversely, LR-based RFE selected DAPSA at baseline (OR 0.85, 95%CI 0.77-0.93), BMI (0.79, 95%CI 0.66-0.95), presence/absence of FMS (0.05, 95%CI 0.004-0.63), axial disease (0.01, 95%CI 0.001- 0.10), HAQ-DI at baseline (0.10, 95% 0.02-0.45) and combotherapy at baseline (0.43, 95%CI 0.19-0.98), all inversely associated with DAPSA remission; only high CRP (mg/l) at baseline (1.08, 95% CI 1.03-1.12) was found to predict 12-month remission. **Table 2** resumes the ORs for each attribute with 95%CI. The performance of XGBoost showed accuracy of 0.97 ± 0.06, recall of 0.96 ± 0.006, precision 0.97 ± 0.006 and AUROC 0.97 ± 0.07 (**Figure 2**). In contrast LR performance was significantly poorer, with accuracy of 0.73 ± 0.09, recall 0.64 ± 0.11, precision 0.85 ± 0.09, AUROC 0.78 ± 0.14 (p<0.0001 for all, **Table 3**). In **Figure 3**, a diagnostic calibration has been plotted for XGBoost after 10-fold isotonic calibration; DAPSA remission roughly happened with an observed relative frequency consistent with the forecast value, showing a suitable calibration curve.

**Table 2:** Odds Ratios of Logistic Regression for Multivariable Analysis after Random Feature Elimination.

	OR	95%CI		p
BMI	0.79	0.66	0.94	0.01
HAQ-DI at Baseline	0.10	0.06	0.45	0.003
Baseline csDMARD	0.43	0.19	0.98	0.045
DAPSA at Baseline	0.85	0.77	0.93	0.001
PASI at Baseline	0.62	0.45	0.85	0.003
CRP at Baseline (mg/l)	1.08	1.03	1.13	0.002
FMS	0.05	0.00	0.63	0.02
Axial disease	0.01	0.00	0.19	<0.001

BMI, Body Mass Index; CRP, C Reactive Protein; DAPSA, Disease Activity in Psoriatic Arthritis; ESR, Erythrocyte Sedimentation Rate; FMS, Fibromyalgia; HAQ-DI, Health Assessment Questionnaire- Disability Index; LEI, Leeds Enthesitis Index; MetSyn, Metabolic Syndrome; PASI, Psoriasis Area Severity Index; SD, Standard Deviation.

**Table 3:** Algorithm performance.

	Cut-off	Accuracy	SD	Recall	SD	Precision	SD	AUROC	SD
LR	0.42	0.73	0.09	0.64	0.11	0.85	0.09	0.78	0.14
XGBoost*	0.82	0.97	0.06	0.96	0.006	0.97	0.06	0.97	0.07

*p<0.0001.

LR, Logistic Regression; SD, Standard Deviation; XGBoost, eXtreme Gradient Boosting.

## Discussion

PsA is a complex disease. Ideally, while the clinical management of patients with only psoriasis and synovitis may seem to be linear, treatment becomes much less immediate when the clinical phenotype encompasses enthesitis, axial disease, and comorbid conditions such as FMS and MetSyn (18). Indeed, because of the heterogeneous and multifaceted presentation, there is a lack of biomarkers for diagnosis and prognosis (19). Hence, the dearth of clinical and serological predictors remains despite the growing knowledge of PsA pathogenesis. Given that, new approaches such as ML may lead to valuable insights. In this study, we retrospectively evaluated the 12 months DAPSA based remission in PsA patients on treatment with SEC and built different statistical models for predicting the achievement of DAPSA remission.

We found a significant decrease in disease activity at 12-month treatment with SEC and remission occurred with a similar frequency as in other Italian and international cohorts (8, 20). Furthermore, SEC was proven to improve LEI, PASI, and BASDAI at 12 months. Interestingly SEC did show a good steroid-sparing effect. We also observed a decrease in HAQ-DI, albeit non-significant, with about a fifth of our cohort achieving a HAQ-DI improvement ≥ 0.35. Such improvement is slightly less relevant than recently reported in a large multicenter Italian study (6). Our results should be interpreted in the light of our patient characteristics, depicting a sample with a long disease duration and beyond the 2^nd^ treatment line for the largest part, both features negatively impacting the quality of life (1). This is may also explain the high rate of patients on steroids at SEC baseline. Interestingly, considering that most of them were anti-TNF therapy inadequate responders, our data support the recent evidence that, in need of a rapid anti-inflammatory effect or bridging of therapies, a trend towards prescribing glucocorticoids in PsA patients still exists (21). We demonstrated that a nonlinear ML approach might outperform linear methods such as LR in predicting 12-month DAPSA remission in patients on SEC, based on similar attribute core sets.

Among features deemed as necessary for prediction, several attributes representative of disease activity in different domains, such as baseline DAPSA, PASI and serum acute phase reactants levels, were identified by both algorithms as negatively associated with 12-month remission. Conversely, the importance of baseline LEI in predicting DAPSA remission was underlined exclusively from the XGBoost algorithm. Of note, enthesitis may be often overlooked as entheseal assessment is not routinely performed in real-life settings (22). Indeed, the XGBoost choice might be expected, considering that enthesitis was associated with a high overall PsA burden in the DANBIO Register (22).

It is noteworthy that both RFE wrappers recognized FMS as one of the most important comorbidity to take into account in managing PsA patients on SEC. FMS had been recently recognized as one of the main predictors of drug discontinuation in PsA patients on TNF inhibitors (12), with almost no PsA patients with comorbid fibromyalgia achieving clinical disease remission upon anti-TNF therapy (11). The negative impact of FMS on PsA disease measures in patients on SEC had not been thoroughly investigated yet; as noticeable at a glance by the effect size measures, such as XGBoost feature importance or LR OR, patients with FMS were less likely to achieve 12-month remission. These findings were consistent with a recent study on a cohort of Spanish PsA patients on SEC treatment, reporting the negative impact of depression on drug survival (23). However, FMS was not investigated as a predictor of drug discontinuation in such settings.

Our study also provided insights into the effectiveness of SEC in PsA patients with axial disease. Corevitas (formerly Corrona) Registry had shown that patients with axial PsA had higher disease activity, reduced quality of life and more impaired physical function and work productivity than those without axial involvement (24). Such evidence might explain why both algorithms found axial disease as a negative predictor of DAPSA remission. In particular, XGBoost recognized such domain as the third most crucial attribute for prediction, underlining the need to routinely assess spine involvement in PsA patients. In this study, overweight patients or patients with obesity having PsA were less likely to achieve DAPSA remission. For each 1-point increase in BMI, the probability of achieving DAPSA remission decreased by 20.3%. This is consistent with a previous report showing in a large prospective cohort that obesity can hinder the clinical response to SEC (11). On the other hand, two recent reports on Spanish and Italian cohorts (6, 23) showed that obese patients were less likely to discontinue SEC and a better response to SEC could be achieved in obese individuals compared with normal-weight patients. Potential unknown confounders may account for such discrepancy. However, when focusing on predictive modelling, we noticed that XGBoost overperformed LR without even considering PsA patients BMI.

Unlike rheumatoid arthritis, where unidimensional relations are feasible, PsA needs a multidimensional approach to build predicting models. The superiority of ML methods considering nonlinear relationships is essential as it suggests that we should refine our *modus cogitandi* when dealing with PsA. Far from being a mechanistic chain of cause and effect, rather than simply switching off a specific pathway, it is conceivable that the biologic agents disrupt and modulates the interplay between components of the clinical phenotype resulting in a new balance of interactions. ML approach has the advantage of addressing the clinical issues of PsA management, considering it as a complex system underlying a multifaceted syndrome. Interestingly, the core set of attributes used for training both LR and ML algorithms includes common predictors. However, traditional linear methods such as LR may not be sensitive to perceiving how the attributes interact at baseline and during SEC treatment. When focusing on predictive modelling instead of statistical inference, tree-based algorithms such as XGBoost seem to be a better choice. Another perk of a ML approach with immediate aftermaths into clinical practice is to implement tools readily available on mobile devices and/or desktops capable of providing probability scores, which may help clinicians better identify SEC-good responders, potentially saving indirect costs due to treatment failure.

Some shortcomings must be acknowledged, such as the retrospective design and the relatively small sample size, even if the applied methods have proven robust also for a small-sized dataset. However, an external validation on an independent cohort is required to adopt the XGBoost algorithm in clinical practice and evaluate the attribute core set in an unbiased manner. Nevertheless, we showed that a ML approach has undisclosed potential for guiding the management of PsA patients on SEC and for unravelling the complex interactions between PsA clinical phenotype and IL-17 blockade.

## Data Availability Statement

The raw data supporting the conclusions of this article will be made available by the authors, without undue reservation.

## Ethics Statement

The study involving human participants were reviewed and approved by the Ethics Committee of the University of Bari (Biopure registry, IRB Approval n.5940, Azienda Ospedaliera Universitaria di Bari). Written informed consent to participate in this study was provided by the participants’ legal guardian/next of kin. The patients/participants provided their written informed consent to participate in this study.

## Author Contributions

VV, FI, and GL conceived the study design, drafted the manuscript, and contributed to discussion. VV performed data analysis and drafted the manuscript. ST supervised machine learning methods. AA, SC, and MM collected the data and contributed to discussion. All authors listed have made a substantial, direct, and intellectual contribution to the work and approved it for publication.

## Funding

This study received funding from Novartis Farma SPA.​ The funder was not involved in the study design, collection, analysis, interpretation of data, the writing of this article or the decision to submit it for publication.

## Conflict of Interest

FI and GL received speaker honoraria from Novartis.

The remaining authors declare that the research was conducted in the absence of any commercial or financial relationships that could be construed as a potential conflict of interest.

## Publisher’s Note

All claims expressed in this article are solely those of the authors and do not necessarily represent those of their affiliated organizations, or those of the publisher, the editors and the reviewers. Any product that may be evaluated in this article, or claim that may be made by its manufacturer, is not guaranteed or endorsed by the publisher.
