# Supplementary material for: A Machine Learning Approach to Predict Remission in Patients With Psoriatic Arthritis on Treatment With Secukinumab
Source: Front Immunol. 2022 Jun 27;13:917939. doi: 10.3389/fimmu.2022.917939 (PMC9271870; doi:10.3389/fimmu.2022.917939)
Supplement: Supplementary file 1 [file Table_1.docx]

**SUPPLEMENTARY MATERIAL**

*Feature Selection*

For classification with small training samples and high dimensionality, feature selection plays an important role in avoiding overfitting problems and improving classification performance. One of the commonly used feature selection methods for small samples problems is the wrapper feature selection using recursive feature elimination (RFE) algorithm [1].

Provided with a model that has feature coefficients (e.g. regression) or importance factors (e.g. trees), RFE starts from all features and greedily eliminates the least important feature. Once all features are removed, the algorithm returns the subset which gives the best performance (*backward selection)*.

RFE can generate different subsets of features based on different criteria. The subset generated in each step will be used to iteratively build a model and train the learning algorithm.

This is achieved by fitting the given machine learning algorithm used in the core of the model, ranking features by importance, discarding the least important features, and re-fitting the model.

**Tree-based RFE.** If the relationship between a feature and the output is suspected to be non-linear, tree-based methods (e.g., decision trees, random forest, and extreme gradient boosting (XGBoost)) can be applied to perform feature selection with low complexity. They can model non-linear relations well and do not require much tuning. Simple decision tree algorithm firstly acquires every feature’s importance according to their classification contribution. Then, the features are sorted from high to low according to their importance. Lastly, the least important feature is eliminated, and the attribute set is used re-train the tree model, obtaining a classification performance using the new feature set. This process continues recursively until the algorithm has reached the minimum number of requested features (i.e. n=1). Then, the attribute core set with the least feature and the best performance is output.

XGBoost is a gradient boosted decision tree that is designed for speed and performance. In this method, new models are created that predict the errors of prior models to make the final prediction. This method runs several times faster than existing state-of-the-art methods on a single machine. It scales the models in distributed or memory limited settings, which is the main factor that leads to the success of XGBoost. The stopping criteria for XGBoost are as follows: First, for each output y_i_, the R^2^ of regression performance is measured with all features as input. The corresponding feature importance is also ranked in terms of information gain, which is the relative contribution of each feature to the full model. The second step is to iterate through the ranked list of input attributes and recursively eliminate attributes from the least important feature to the most important feature. After each elimination, the XGBoost model is built with the remaining features, and the corresponding R^2^ score is returned. Finally, with the plot of feature number versus R^2^ score, the elbow method is used to select the optimal number of features. Elbow method identifies the point at which adding more features does not improve the R score.

Tree algorithms are capable of detecting non-linear effects or interactions. Therefore, these methods have the potential to fit into data with significantly fewer features [2].

**Logistic regression-based RFE**. A RFE using logistic regression starts including all covariates into a model In particular, a full logistic regression model is initially built with all of the input features, which can return both the coefficients of the linear function *fi*, and the p-value of each feature. The p-value for each input tests the null hypothesis that the coefficient is equal to zero (i.e., not related to the output). A low p-value (less than threshold 0.05) indicates that the null hypothesis should be rejected, and this feature will be a meaningful input to the ordinary lease square (OLS) model. Conversely, a larger (insignificant) p-value suggests that changes in the feature are not associated with changes in the output and should be excluded from the model. With the full model built, if the maximum p-value is greater than the threshold 0.1, the corresponding least significant feature (with the biggest p-value) is removed, and the OLS model is rebuilt with remaining. This feature elimination process is repeated until no improvement is observed on the removal of features, i.e., all of the remaining features have a p-value less than threshold α= 0.1.

*Cross.validation*

Cross-validation is a [model validation](https://en.wikipedia.org/wiki/Model_validation) technique for estimating the expected error of  [the model on an unseen data set of similar distribution.](https://en.wikipedia.org/wiki/Generalization_error) In *k*-fold cross-validation, the original sample is randomly partitioned into *k* equal sized subsample. Only one of the *k* subsamples is retained as validation data for testing the model, and the remaining *k* − 1 subsample is used as training data. The cross-validation process is repeated *k* times, with each of the *k* subsamples used exactly once as the validation data. The *k* results can then be treated as samples of the model’s error distribution. The perk of this method is that all observations are used for both training and validation, and each observation is used for validation exactly once. Five or ten are the values commonly used for the meta parameter *k.* (3)

*Bayesian Ridge Conditional Imputation on Scikit.learn Iterative Imputer*

A multivariate imputer estimates each feature from all the others. Deploying this method is considered a powerful strategy for imputing missing values by modeling each feature with missing values as a function of other features in a round-robin fashion [3].Scikit.learn Iterative Imputer uses Bayesian Ridge regression as default. There exist several strategies to perform Bayesian ridge regression. The Scikit.learn implementation is based on the algorithm described by Tipping [4] where updates of the regularization parameters are done as suggested by MacKay [5]. Iterative Imputer has proven to be the more accurate method of imputation for obstetrics and ginechology datasets [6].

*Hyperparameters fine-tuning*

A model hyperparameter is a characteristic of a model that is external to the model and whose value cannot be estimated from data.

Grid search is the process of performing hyperparameter tuning in order to determine the optimal values for a given model. This is significant as the performance of the entire model is based on the hyperparameter values specified.

In brief, for each algorithm, analysts define a grid consisting of several values to test for any hyperparameter of interest. The grid search performs a K-fold cross-validation splitting a dataset in K partitions and then searching for each split the combination leading to the best performance in a grid of hyperparameters[3].

The hyperparameter grids for each algorithm have been shown below:

Logistic Regression [3]:

- Solver: ‘lbfgs’, ‘liblinear’ // Selected: ‘lbfgs’

XGBoost[3]:

- Number of trees in the forest: (100, 500 ,100) // Selected: 500
- Maximum depth of the tree: (3, 5, 10)// Selected: 3

**REFERENCES**

1. Zeng X, Chen Y, Tao C, Alphen Dv. Feature Selection Using Recursive Feature Elimination for Handwritten Digit Recognition. *2009 Fifth International Conference on Intelligent Information Hiding and Multimedia Signal Processing* 2009. p. 1205-8.

2. Kamel E, Sheikh S, Huang X. Data-driven predictive models for residential building energy use based on the segregation of heating and cooling days. *Energy*. 2020;206:118045.

3. Buitinck L, Louppe G, Blondel M, Pedregosa F, Mueller A, Grisel O, et al. API design for machine learning software: experiences from the scikit-learn project. 2013:arXiv:1309.0238. Accessed: September 01, 2013.

4. Tipping ME. Sparse bayesian learning and the relevance vector machine. *J Mach Learn Res*. 2001;1:211–44.

5. MacKay DJC. Bayesian Interpolation. In: Smith CR, Erickson GJ, Neudorfer PO, eds. *Maximum Entropy and Bayesian Methods: Seattle, 1991*. Dordrecht: Springer Netherlands; 1992. p. 39-66.

6. Altukhova O. Choice of method imputation missing values for obstetrics clinical data. *Procedia Computer Science*. 2020;176:976-84.
